# Development and preliminary testing of the psychosocial adjustment to hereditary diseases scale

**DOI:** 10.1186/2050-7283-1-7

**Published:** 2013-04-30

**Authors:** Kathy E Watkins, Christine Y Way, Deborah M Gregory, Holly M LeDrew, Valerie C Ludlow, Mary Jane Esplen, Jeffrey J Dowden, Janet E Cox, G William N Fitzgerald, Patrick S Parfrey

**Affiliations:** Centre for Nursing Studies, Eastern Regional Integrated Health Authority, St. John’s, NL Canada; Clinical Epidemiology Unit, Faculty of Medicine, Memorial University of Newfoundland, St. John’s, NL Canada; School of Nursing, Memorial University of Newfoundland, 300 Prince Philip Drive, St. John’s, NL A1B 3V6 Canada; Eastern Regional Integrated Health Authority, St. John’s, NL Canada; Western Regional School of Nursing, Western Regional Integrated Health Authority, Corner Brook, NL Canada; Department of Psychiatry, Faculty of Medicine, University of Toronto, Toronto, ON Canada; Newfoundland and Labrador Centre for Health Information, St. John’s, NL Canada; Division of Surgery, Charles S. Curtis Memorial Hospital, St. Anthony, NL Canada

**Keywords:** Lynch syndrome, Hereditary diseases, Genetic testing, Psychometric testing

## Abstract

**Background:**

The presence of Lynch syndrome (LS) can bring a lifetime of uncertainty to an entire family as members adjust to living with a high lifetime cancer risk. The research base on how individuals and families adjust to genetic-linked diseases following predictive genetic testing has increased our understanding of short-term impacts but gaps continue to exist in knowledge of important factors that facilitate or impede long-term adjustment. The failure of existing scales to detect psychosocial adjustment challenges in this population has led researchers to question the adequate sensitivity of these instruments. Furthermore, we have limited insight into the role of the family in promoting adjustment.

**Methods:**

The purpose of this study was to develop and initially validate the *Psychosocial Adjustment to Hereditary Diseases (PAHD)* scale. This scale consists of two subscales, the *Burden of Knowing (BK)* and *Family Connectedness (FC)*. Items for the two subscales were generated from a qualitative data base and tested in a sample of 243 participants from families with LS.

**Results:**

The Multitrait/Multi-Item Analysis Program-Revised (MAP-R) was used to evaluate the psychometric properties of the PAHD. The findings support the convergent and discriminant validity of the subscales. Construct validity was confirmed by factor analysis and Cronbach’s alpha supported a strong internal consistency for BK (0.83) and FC (0.84).

**Conclusion:**

Preliminary testing suggests that the PAHD is a psychometrically sound scale capable of assessing psychosocial adjustment. We conclude that the PAHD may be a valuable monitoring tool to identify individuals and families who may require therapeutic interventions.

## Background

Lynch syndrome (LS) is an autosomal dominant disease characterized by the development of colorectal (CRC) and extracolonic cancers (Stuckless et al. 2007). Individuals living with LS may be faced with cancer onset in themselves and other family members, lifelong cancer screening, extensive treatment regimes and early deaths of family members. Confirmation of LS through predictive genetic testing can bring a lifetime of uncertainty to an entire family as members adjust to living with an indeterminate or evolving disease state. The research base on how individuals and families adjust to genetic-linked diseases following predictive genetic testing has increased our understanding of short-term impacts but gaps continue to exist in knowledge of important factors that facilitate or impede long-term adjustment.

In studies focusing on the impact of genetic-based diseases, the adjustment construct assumes many forms. Psychological/psychosocial adjustment is used interchangeably with psychological/psychosocial functioning, impact, distress, consequences and outcomes, among others. What is evident from a review of the scientific literature is a lack of consensus on how psychological adjustment is defined and operationalized (Wechsler & Sanchez-Iglesias 2012).

Quantitative studies that focus on hereditary cancer have primarily assessed short-term psychological functioning (i.e., cancer specific distress, anxiety, and depression) by using such standardized scales as the State-Trait Anxiety Inventory (Aktan-Collan et al. 2001; Claes et al. 2004; Collins et al. 2007; Esplen et al. 2001; Esplen et al. 2003; Esplen et al. 2007; Meiser et al. 2004), Impact of Events Scale (Aktan-Collan et al. 2001; Collins et al. 2007; Esplen et al. 2001; Esplen et al. 2003; Esplen et al. 2007; den Heijer et al. 2011), Hospital Anxiety and Depression Scale (Collins et al. 2007; Meiser et al. 2004; den Heijer et al. 2011), and the Center for Epidemiologic Studies for Depression Scale (Esplen et al. 2001; Esplen et al. 2003; Esplen et al. 2007). The evidence suggests that individuals who are part of LS families are not distressed (intrusive thoughts about cancer, anxiety and depression) in the short-term post-genetic testing. Prospective studies monitoring changes in psychological functioning during genetic testing show slight elevations in carriers distress levels immediately post-testing which return to baseline levels within a year, but decrease immediately for non-carriers and remain relatively stable over time (Aktan-Collan et al. 2001; Claes et al. 2004; Esplen et al. 2001; Murakami et al. 2004). Investigations of impact for longer periods revealed no differences in psychosocial outcomes between carriers and non-carriers at three (Collins et al. 2007; Foster et al. 2007) or five years post-testing (van Oostrom et al. 2003). The conclusion of meta-analyses and literature reviews is that genetic testing for hereditary cancer causes minimal psychological consequences (Bleiker et al. 2003; Braithwaite et al. 2006; Heshka et al. 2008; Meiser 2005).

Absent from this quantitative research base is prospective data on long-term psychosocial adjustment. Specifically, there is minimal consideration of the psychosocial and emotional impact of living with hereditary cancer, personal and family challenges over time and the role played by family functioning and supports in reducing the impact of hereditary cancer and facilitating adjustment. In 2004, our research team administered a battery of standardized and researcher-developed scales to a convenience sample of 120 carriers and non-carriers from LS families in Newfoundland and Labrador at different times post-genetic testing (i.e., 0.1 to 9.2 years). Baum and colleagues theoretical model of stress and adaptation (1997) (Baum et al. 1997), previously described by Esplen et al. (2007) (Esplen et al. 2007), was used to guide data collection. Table 1 presents a summary of the objectives, methods and select findings of this initial survey. Study findings revealed that most respondents were not psychologically distressed (anxious, depressed, intrusive and avoidant thoughts) from being involved in genetic testing for LS, did not convey worry/concern about cancer risk for the self/others, were part of healthy functioning families with adequate internal strengths, were satisfied with available social supports, relied equally on emotion-focused and problem-focused coping, and were satisfied with valued aspects of life (family, health & functioning, psychological spiritual and social/economic). Although most individuals seemed well adjusted, a subgroup had elevated distress levels, compromised family functioning and lower quality of life.

**Table 1:** **Objectives, instruments used and results of two preliminary studies undertaken prior to the current study**

Study	Objectives	Instrumentation	Results
Phase I: Survey	1) to investigate psychosocial and behavioral impact of genetic testing (GT) process for at-risk individuals in LS families	Standardized scales (Impact of Events Scale (Horowitz et al. 1979), Centre for Epidemiologic Studies Depression Scale (Radloff 1977), State Trait Anxiety Inventory (Spielberger 1983), McMaster Family Assessment Device (Epstein et al. 1983), Family Hardiness Index (McCubbin et al. 1996), Quality of Life Index (Ferrans & Powers 1992), Social Support Questionnaire (Sarason et al. 1987), Ways of Coping Questionnaire (Lazarus & Folkman 1984)); researcher-developed items (medical history, worry/concerns, demographics, cancer experiences, reaction to & disclosure of results, screening & healthy living)	*Sample characteristics:*
			- mean age of 47.4 (SD = 12.9), range 22 to 78 years
			- female (57.5%), carriers (51.7%) of intron 5 splice site mutation (93.3%) and unaffected (77.5%)
	2) to examine key factors (i.e., age, gender, education, supportive relationships, familial & personal cancer history, CRC knowledge, satisfaction with GT decision, time since GT) associated with difficulties in psychosocial and behavioral adjustment (reaction to GT results, perception of risk, willingness to disclose and to whom) in individuals affected/unaffected with cancer		- average of 6 years post-genetic testing
			*Key findings:*
			- over 33% had moderate to severe avoidance/intrusive thoughts post-GT;
			- small percent above clinical cut-off score for depression and anxiety
			- small percent with quality of life issues and lower family functioning (role execution & communication
			- no significant impact for time since GT, gender, age, carrier or cancer status
Phase II: Qualitative	1) to explore meanings of genetic testing for individuals at risk for colorectal and related-cancers in LS families	Semi-structured interviews focused on: familial cancer experiences (exposure in close/distant members, first aware of hereditary link, perceived risk for self, screening/healthy living motivation) and pre/post GT (decision-making pre and post testing, experience with genetic counseling, reaction to GT results, understanding risk for self/others, impact on family, role/importance of supports, adjusting to status & experiences with health care	*Constructs:*
			- Living in families with a strong history of hereditary cancer (familial cancer context & emergence of hereditary link)
	2) to understand psychosocial and behavioral impact of genetic testing for carriers and non-carriers of LS		- Becoming aware of genetic testing and living the process (decision-making, reactions to results, understand risk, supportiveness of genetic counselors, disclose results)
	3) to use emergent data to improve existing counseling programs		- Struggling to adjust (personal/family challenges, family dynamics/support, barriers/facilitators of adjustment)

There is additional support from the literature that a small, but significant, group of individuals experience adjustment problems and may be classified as having borderline distress (Esplen et al. 2007; van Oostrom et al. 2003; Heshka et al. 2008; Meiser 2005). Problems with psychological functioning may negatively impact long-term adjustment, particularly adherence to recommended screening protocols crucial for the prevention and early detection of cancer. Importantly, the evidence suggests that individuals with greater social supports and who belong to families with open communication are more likely to follow recommended protocols (Johnson et al. 2002; Keller et al. 2002; McCann et al. 2009), have less psychosocial distress (Claes et al. 2005; Loader et al. 2002; van Oostrom et al. 2007) and adjust better over the long-term (den Heijer et al. 2011).

With the sensitivity and specificity of standardized scales for detecting and monitoring psychosocial adjustment in this population questioned (Claes et al. 2004; Bleiker et al. 2003), Read, Perry and Duffy (Read et al. 2005) developed the Psychological Adaptation to Genetic Diseases (PAGIS) scale to evaluate the efficacy of genetic counseling and identify individuals requiring additional support. These researchers propose that psychological adaptation to genetic information is a multidimensional phenomenon comprised of non-intrusiveness, support, self-worth, certainty and self-efficacy. While the PAGIS demonstrated acceptable internal consistency and content validity in preliminary testing, there is no further reference to its use in subsequent studies. The Multidimensional Impact of Cancer Risk Assessment (MICRA) questionnaire (Cella et al. 2002) was developed to measure positive and negative responses to genetic testing for cancer. The MICRA was initially validated among women at risk for breast cancer but, to our knowledge, has not been used in subsequent studies. Despite these disease-specific scales, there is no empirical evidence suggesting that they are capable of monitoring how well individuals adjust to genetic-based diseases in the short-and long-term (Biesecker & Erby 2008).

Critical appraisal of the research evidence on adjustment challenges for LS families from studies using quantitative versus qualitative methodologies can lead to very different conclusions. Reliance on qualitative methods helps researchers identify areas of psychosocial impact that have implications for affective and behavioral outcomes. The evidence suggests that certain individuals have difficulty adjusting in the short- and long-term following confirmation of hereditary cancer (Bartuma et al. 2012; Carlsson & Nilbert 2007; Stermer et al. 2004; Watkins et al. 2011; Hamilton et al. 2009), feel burdened about communicating genetic risk information to family members (Hamilton et al. 2009), worry about cancer risk in others (Bartuma et al. 2012; Carlsson & Nilbert 2007), perceive that health care system supports post-genetic testing are inadequate (Stermer et al. 2004; Watkins et al. 2011), struggle to adhere to recommended screening protocols (Stermer et al. 2004; Watkins et al. 2011) and experience difficulty in coping with cancer in the self/others (Carlsson & Nilbert 2007).

Following the 2004 survey, our research team designed a grounded theory study to explore the meaning of genetic testing for individuals (n = 39) in LS families and develop a greater understanding of psychosocial and behavioral impacts for confirmed carriers and non-carriers. Data collection spanned the years 2004 to 2007. Purposive samples were recruited from 15 family groupings: a) 2004 survey respondents with an interest in further research (n = 22), b) additional individuals from families with the intron 5 splice site mutation to augment evolving family, carrier/non-carrier or affected/non-affected themes (n =10) and, c) individuals from families with the more recently identified exon 8 deletion to ensure comparability of experiences with intron 5 splice site mutation families (n = 7). Details on the sample and data analysis have been described elsewhere (Watkins et al. 2011). Semi-structured schedules guided data collection via face-to face interviews. A second interview confirmed the interpretive summaries constructed from each transcript, augmented gaps in the data and corroborated conceptual categories and properties. Table 1 summarizes study objectives, methods and key findings.

The conceptual model *“Confronting and Accepting the Challenges of Living in Families with Genetic-Linked Diseases”* emerged from analysis of the qualitative data. The model broadly conjectures that the situational and experiential contexts are important forces influencing how well individuals accept the hereditary link to cancer, are motivated to become involved in genetic testing, and adjust to living with a confirmed presence of LS in the family in the short- and long-term. The *struggling to adjust* construct focuses on psychosocial and behavioral adjustment in LS families. The findings suggest that while most individuals acknowledge the importance of knowing about their cancer risk, some are burdened by having to manage LS over time (i.e., struggle to adhere to recommended screening) and having to deal with cancer episodes in the self and/or others. Importantly, the impact of LS is not limited to carriers but extends to all family members. Family functioning and openness of communications seem critical in helping individuals deal with the ongoing challenges. Finally, the findings provide further support for the premise that some individuals in these families experience difficulty adjusting in the short- and long-term and, at times, struggle to effectively manage their disease.

Based on the research literature and quantitative and qualitative findings from the two projects conducted by the research team, it was concluded that reliable and valid clinical tools capable of identifying subgroups of individuals, as well as their families, who may be at-risk for psychosocial and emotional challenges post-genetic testing are needed for use in genetics clinics. Monitoring tools are needed to assess adjustment to LS (i.e., positive affect and well-being, motivation to follow recommended protocols and modify health behaviors, and the buffering impact of supports). It was also evident from the literature and our findings that health care providers tend to not only have limited insight into the extent of individual and family burden posed by genetic-based diseases but also fail to understand the level of support that might be needed to mitigate long-term effects.

In summary, emphasis on short-term outcomes, without thorough consideration of the social and familial contexts, can limit our understanding of long-term psychosocial adjustment. We argue that adjustment to hereditary cancer is broader than psychological outcomes and is an evolving process that ebbs and flows in response to changing personal and family experiences in the management of long-term cancer risk and emergence of cancer in the self and/or others. Personal and/or family experiences can facilitate or impede adjustment.

### Purpose

The purpose of the current study was to develop and initially validate a tool for monitoring long-term psychosocial adjustment. Using the data generated from a grounded theory study, the Psychosocial Adjustment to Hereditary Diseases (PAHD) scale was developed as part of an ethically approved program of research. The PAHD is designed to assess the personal and family burden of LS and the perceived role of family in buffering its impact. The specific objectives for this component of the larger project are to: a) test the feasibility of using the PAHD scale under variant conditions, b) reduce item numbers, c) validate subscale and overall scale structure, and d) examine scaling (rating) methods.

## Methods

The study was conducted in three phases. Phase I consisted of item generation and refinement. Phase II consisted of a pilot study designed to generate data for preliminary assessment of the psychometric properties of the PAHD scale. Phase III was designed to generate additional data to facilitate final item selection and initial scale validation.

### Phase I: scale development

Interview transcripts from the grounded theory study provided the data base for scale development. The grounded theory method facilitated theoretical construct identification in such a manner that operational indicators defining the properties of each construct could be used to generate items. Initially, data matrices were created for the struggling to adjust construct by collating all data from the interviews into relevant descriptors of properties and re-writing the text until a clear decision trail emerged. Two dominant themes emerged from these analyses – one focusing on psychosocial adjustment, and the other on behavioral adjustment. The psychosocial adjustment data matrix provided the content for item generation for the PAHD.

The approach taken to item generation and refinement consisted of several steps which are summarized in Table 2. The first step involved item generation and refinement. The focus was on identifying potential stems, reducing the number of stems and reworking and finalizing the text. The items were grouped into two subscales based on theoretical content. The first subscale dealt with personal burden issues (i.e., psychosocial distress and emotional well-being), and the second with family dynamics and the importance of openness and supports. At the second step, efforts focused on selecting the best rating scale format to use with this population. Following consideration of multiple selection options, the research team decided to use one rating scale (not at all, a little bit, moderately, quite a bit, extremely). The fifth and final steps focused on assessing the scale’s readability and subjecting it to content validation. The readability level of the PAHD was at an acceptable level and genetic counselors and individuals from LS families validated the content of the PAHD, as well as the usefulness of the rating scale.

**Table 2:** **PAHD Scale development**

Item stem identification	A four-member research team was responsible for item generation and refinement. Initially, the team became immersed in the data matrices of the *struggling to adjust* construct. Independent raters created a profile of frequency and priority ratings of construct properties and descriptors (e.g., dwelling on carrier status, positive outlook, concern for young family members, importance of openness, strain on relations, emotional burden of suffering & death) by participant and group. Team members used these profiles to generate item stems for 5 groups and the principal investigator validated the process. At this stage, the team had 59 potential items.
Item stem reduction	Multiple drafts of items for the scale were reviewed and modified by the researchers. Team meetings were held frequently to collate, prioritize and refine item stems for potential scale inclusion (emphasis on conciseness, avoidance of negative wording, ambiguous terminology, jargon, value-laden words and double-barreled questions). A final set of 17 items were identified for potential inclusion in the PAHD scale.
Rating scale development	Initial rating scales focused on the frequency of occurrence (never, rarely, sometimes, often, or almost always), and ‘the importance/difficulty/receptiveness of’ or ‘how satisfied/concerned/confident/certain one was with’ select events/situations (not at all, a little bit, moderately, quite a bit, extremely). The multiple selection options made things cumbersome and confusing. The decision was made to rework the items and use one rating scale. Despite recognizing that a 5-point scale might not be sufficient for maximum reliability, the group consensus was that it would be difficult to devise unambiguous additional ordinal adjectives.
Scale readability	Several tools (i.e., Flesch-Kincaid Grade Level and Flesch Reading Ease, Fog index and SMOG) were used to assess the PAHD’s reading level at less than or equal to Grade 10. Although a grade less than 10 is recommended to ensure maximum reading ease and material comprehension, the PAHD is developed to assess the experiences of individuals who have had predictive DNA testing. These individuals have had repeated exposure to terms such as LS, hereditary non-polyposis colorectal cancer, carriers/non-carriers, inherited, generations, genetic and geneticist/genetic counselor. These polysyllabic words and others are used frequently throughout the scale which does increase the final readability score.
Content validation	*First* ***,*** two genetic counselors (GCs) who work with individuals during the genetic testing process reviewed the PAHD. A brief written synopsis of the conceptual model and construct definitions, along with a copy of the scales, were given to the GCs to prepare them for this task. Input was requested on item content relevancy (extremely, moderately, slightly, or irrelevant) in terms of its ability to measure the properties of targeted constructs, and effectiveness (very, moderately, poorly or not at all effective) of the 5-point Likert rating scale for ease of item rating. Minor changes to select items were made based on their recommendations. *Second*, the PAHD was administered to individuals (carrier & non-carrier) who had participated in the survey and qualitative studies. Respondents were asked to comment on item clarity/relevancy, and rating scale usefulness. No changes were made at this stage.

### Phase II: pilot study

Using a descriptive correlational design with longitudinal components the PAHD scale was initially tested in individuals from LS families. The approach to scale testing was based on the work of Ware and Gandek (Ware & Gandek 1998), a method used by others (Radwin et al. 2003; Radwin et al. 2005).

#### Methods

The pilot study was designed to assess the integrity of subscale and scale structures, item clarity and difficulty, time required for completion and the feasibility of using different administrative methods. It also provided data for a preliminary assessment of the PAHD scale. Following creation of a descriptive profile for each item (i.e., frequencies, means, standard deviation, skewness and missing data), a correlation matrix was generated and the strength and significance of inter-item correlations assessed. A summary table was constructed of inter-item correlations falling within set cutoff ranges (i.e., >.40 and .30 to .40) which was the primary basis for initial subscale item selection. The final steps included factor analysis and reliability analysis using Cronbach’s alpha.

#### Population and sample

The target population was individuals at 50% risk for inheriting LS who had participated in genetic testing and informed of their carrier status. Survey respondents were recruited from families attending the Provincial Medical Genetics Program of Newfoundland and Labrador (PMGP-NL). Three large pedigrees with MSH2 mutations on intron 5, exon 8 or exon 4 to 16 have been identified with 272 carriers and 295 non-carriers confirmed and entered into a Cancer Screening Data Base. This data base provided the resource for subject recruitment for the pilot study which occurred between February and June of 2008. Of the 120 individuals contacted, 75 (45 carriers and 30 non-carriers) completed the survey, resulting in a 62.5% response rate.

#### Procedure

Ethical approval of the study protocol was granted by the Human Investigation Committee, Faculty of Medicine, Memorial University as well as Eastern Health where the PMGP-NL is located. Telephone contact was initiated with potential respondents to inform them about the study and ascertain their willingness to receive additional information. Consenting individuals were forwarded packages consisting of a cover letter, a brief summary of the study, two consent forms and the survey instrument. Following receipt of consent, a follow-up telephone call was made to determine the preferred mode of participation (face-to-face, telephone or self-administered) and to schedule a mutually agreed upon time for survey completion.

#### Preliminary results

Importantly, data completeness was similar for all three methods of PAHD administration, indicating that it is possible to administer this scale under variant conditions. Preliminary findings indicated that the two subscales appeared to be sensitive enough to measure a range of factors influencing psychosocial adjustment. For most items, there was evidence of fair spread across the response choices. Although factor analysis indicated that item sampling was less than desired, no further analyses were pursued until further subject recruitment.

#### Post-pilot findings

Following recruitment of additional respondents, the PAHD subscale structure was reexamined. The items comprising the two subscales were merged with items from the subscales of the Hereditary Diseases and Genetic Testing (HD-GT), a second scale developed by the research team to assess the impact of the genetic testing process (pre, during and post receipt of results), and a correlational matrix generated. It was anticipated that this approach would help the research team determine if meaningful divisions existed between the subscales of the HD-GT dealing with psychological and emotional issues from engaging in genetic testing compared to those of the PAHD which focus on assessment of more long-term effects. The correlation matrices confirmed the uniqueness of the PAHD subscales and identified additional items not loading on any HD-GT subscales but theoretically similar in content to PAHD items.

The final PAHD scale (Appendix 1) contained two subscales with 17 items (Table 3). The *Burden of Knowing (BK)* and *Family Connectedness (FC)* subscales are in line with the psychosocial and emotional component of the construct *struggling to adjust*. The conceptual definition highlights the importance of capturing: a) the perceived personal and/or family burden following confirmation of LS and b) the role played by family supports in promoting status acceptance and buffering the impact of challenges posed by the disease. The BK scale is comprised of 10 items that recognize the personal and family aspects of adjustment to hereditary cancer with higher scores reflecting lesser burden. Additional items from the HD-GT scale address how the stress of cancer in younger family members may impact family relations (BK19_R) and how regular screening may heighten cancer worries (BK20_R, BK27_R).

**Table 3:** **Item descriptive statistics for Burden of Knowing (BK) and Family Connectedness (FC) scales (n = 243)**

Scale & Items	X	SD	Missing (%)	Response Values Frequency				
				0	1	2	3	4
**Burden of Knowing (BK)**	24.8	8.4	9.5%					
• Dwelling on carrier status (BK11_R)	3.1	1.1	0.8	5	24	36	65	111
• Difficulty modifying screening regime (BK14_R)	2.8	1.4	1.6	28	17	41	34	119
• Concerns with non-acceptance by others (BK15_R)	3.4	1.2	2.5	13	14	12	27	171
• Difficulty dealing with young people (BK17_R)	1.8	1.4	3.3	59	43	59	28	46
• Worry about young people’s future (BK18_R)	1.4	1.3	1.2	80	58	49	39	14
• Stress of cancer alters family relations (BK19_R)	2.8	1.3	1.6	17	33	39	40	110
• Screening reminder of personal risk (BK20_R)	2.2	1.5	1.2	50	37	44	39	70
• Concerns about impact on family relations (BK24_R)	3.3	1.2	0.4	13	15	24	25	165
• Worry about burden of cancer on family (BK25_R)	2.3	1.4	0.8	30	44	54	49	64
• Screening heightens cancer worry (BK27_R)	1.9	1.5	0.8	63	46	41	45	46
**Family Connectedness (FC)**	20.4	5.6	4.5					
• Encourage young people to talk about cancer (FC16)	3.0	1.1	1.2	10	18	49	60	103
• Feeling supported facilitates acceptance (FC21)	2.9	1.2	0.4	13	21	41	77	90
• Easy to seek help from family (FC22)	3.0	1.2	0.8	13	18	34	67	109
• Important to openly discuss family cancer (FC23)	3.4	0.8	0.4	0	8	27	62	145
• Caring for others promotes personal acceptance (FC26)	2.3	1.4	1.6	37	31	50	66	55
• Relieved by availability of genetic testing (FC28)	2.9	1.2	1.6	10	21	42	66	100
• Supportive others promotes healthy behaviors (FC29)	3.0	1.1	0.4	11	16	33	78	104

Comparatively, the seven-item FC scale assesses family connectedness with higher scores reflecting the importance of having open discussions and access to resources to handle the challenges posed by LS. Additional items from the HD-GT scale address feelings of relief concerning the availability of genetic testing (FC28) and the role of supportive others in promoting acceptance of healthy behaviors (FC29). Two items dealing with emotional well-being (BK12) and not dwelling on the hereditary cancer (BK13) failed to load on either subscale but were retained as test items for future scale administrations.

### Phase 3: initial validation

Ongoing recruitment and data collection continued between July 2008 and July 2010. Data were collected by face-to-face interviews, telephone interviews and self-administered surveys. Of the additional 253 individuals contacted, the scale was administered to another 168 participants. In total, 373 individuals agreed to receive study materials during the two phases giving a total sample size of 243 (140 carriers and 103 non-carriers of LS) and a response rate of 65.1%.

Study respondents were mostly females (63.8%) and from families with a confirmed MSH2 gene mutation (92.6%). Of the MSH2 mutations (intron 5 splice site, exon 8 deletion or exon 4–16 deletion), the dominant type was the intron 5 splice site (62.1%). The remaining participants had mutations in either MLH1 (6.6%) or MLH6 (0.8%). The mean age was 48.80 (SD =13.60), with a range of 19 to 83 years. Most participants were carriers (57.60%) but unaffected by cancer at the time of the study (72.80%). Although study respondents and non-responders were similar with regard to gender (*χ*^2^ (1, N=) = 2.08, p > 0.05), non-responders tended to be non-carriers (*χ*^2^ (1, N=) = 4.79, p < 0.05) and younger (t (361) = −2.63, p < 0.01) than respondents.

### Data analysis

Data were coded and entered into the Statistical Package for the Social Sciences (SPSS) for analysis. Descriptive statistics were used to create a profile of respondents’ scores on all study scales. The Multitrait/Multi-Item Analysis Program-Revised (MAP-R) assessed how well the PAHD met Likert scaling assumptions (Ware et al. 1997). At the first step, the assumption concerning the appropriateness of using particular items to create a summative score (approximate equivalence of means and variances, use of all response choices in the rating scale, amount of missing data, and approximate symmetry in response distribution) were assessed. At the second step, a multitrait/multi-item correlation matrix was generated to assess three additional assumptions (linearity, item-convergent validity and item-discriminant validity). At the third step, subscale scores were assessed in terms of ceiling and floor effects, approximate symmetry, internal consistency and inter-correlations. Finally, factor analysis examined the construct validity of the 17-item PAHD scale. The appropriateness of the factor analytic model was tested using the Kaiser-Meyer-Olkin (KMO) measure of sampling adequacy and Bartlett’s test of sphericity. Principal component and maximum likelihood analysis were the factor extraction methods. The scree test was used to determine the number of factors to retain. The preferred rotation method was orthogonal using varimax rotation.

## Results

### Data quality and item-level summated scale assumptions

#### Data quality

Item descriptives for the PAHD scale are displayed in Table 3. Missing data for individual items were random and minimal, ranging from 0.4% to 3.3%. Although there is no consensus on what constitutes extensive missing data (from 10%-40%) on any given item or variable, it is generally agreed that what is more important is whether the pattern is systematic or random in nature (El-Masri & Fox-Wasylyshyn 2005).

The majority of respondents had complete data for the two subscales. The percent of respondents with complete data ranged from 90.5% for BK to 95.5% for FC (data not shown). The minimum and random amount of missing data for this study suggests that overall the scale items were not difficult to understand or interpret (Ware & Gandek 1998).

All response choices were used for most items (94.1%). The data also depict variability across the rating scale and approximate a symmetrical distribution. The subscale items with minimal to no use of certain response choices were expected. For example, most individuals are expected to attach high importance to having family members talk openly about the high cancer risk (FC23).

#### Item-level scaling assumptions

Items means and standard deviations within each subscale are approximately equivalent (Table 3). There are important exceptions, however, which require further elaboration. In the BK subscale, items 17, 18 and 27 have lower mean scores and greater variance than the remaining items. This finding is expected given that these items are more focused on personal worries and interaction difficulties. The higher mean scores and lower variances observed for items 11, 15 and 24 were also expected since their content focuses on the personal and family implications of knowing one’s carrier status and dealing with LS. Similarly, the higher score and lower variance observed for item 23 of the FC subscale was also expected as most individuals attach importance to open discussion of high cancer risk among family members.

### Scale level assumptions

#### Item internal consistency

Table 4 outlines Pearson item-scale correlations corrected for item overlap (Ware & Gandek 1998; Howard & Forehand 1962). Item-scale correlations were used to examine the relationship of each item to its hypothesized scale (i.e., internal consistency). Correlations for all items within their respective scales are larger than correlations between items and competing scales. In addition, all item-scale correlations are 0.42 or larger indicating a substantial and satisfactory item internal consistency (Ware & Gandek 1998).

**Table 4:** **Factor scores and final item to scale correlations**

Scale item	Factor 1	Factor 2	BK^§^	FC^§^
BK11_R	**.620**	-.121	0.58*	-0.27
BR14_R	**.555**	-.051	0.45*	-0.19
BK15_R	**.516**	-.080	0.46*	-0.21
BK17_R	**.531**	-.150	0.49*	-0.27
BK18_R	**.562**	-.412	0.55*	-0.52
BK19_R	**.520**	-.160	0.48*	-0.29
BK20_R	**.583**	-.218	0.55*	-0.33
BK24_R	**.473**	-.055	0.42*	-0.18
BK25_R	**.612**	-.188	0.56*	-0.34
BK27_R	**.533**	-.209	0.50*	-0.33
FC16	-.263	**.524**	-0.38	0.46*
FC21	-.103	**.769**	-0.32	0.70*
FC22	.001	**.706**	-0.20	0.59*
FC23	-.180	**.798**	-0.39	0.73*
FC26	-.237	**.503**	-0.37	0.51*
FC28	-.162	**.537**	-0.30	0.48*
FC29	-.180	**.600**	-0.34	0.58*

Abbreviations: *BK* = Burden of knowing, *FC* = Family connectedness.

Extraction Method: Maximum likelihood; Number of factors to retain: Scree test; Rotation method: Varimax.

^**§**^ Item-scale correlation corrected for overlap (relevant item removed from its scale for correlation). ^*^Denotes item correlations with hypothesized scales.

#### Equality of item-scale correlations

This assumption addresses the proximity of values for all item-scale correlations within a hypothesized scale. The best scale contains item-scale correlations that are roughly equal and ideally fall within the 0.40 to 0.70 range (Ware & Gandek 1998). The reader is again referred to the corrected item-total correlations for individual items and their subscales in the columns with asterisks in Table 4.

For the majority of items in the two subscales, the corrected-item total correlations fall within an acceptable range. There are some exceptions however. The items that appear to be contributing more to their various scales than other items include items 21 and 23 of the FC subscale. These items deal with emotional content which may be responsible for the observed discrepancies. This finding is expected to a degree since item content is focused on the importance of feeling supported by family/friends in coming to terms with being a carrier/non-carrier and the importance of family members openly discussing the cancer risk.

#### Item discriminant validity

This assumption examines the strength of item correlations with other scales with the objective that each item has a stronger correlation with its hypothesized scale than with other related scales. Study findings are summarized in Table 4. Four score categories (−1, -2, +1 or +2) are possible for each test with the standard error of correlation setting the criterion. Values of −1 and −2 indicate that an item has failed the test of item discriminant validity. In this study, all item scale discriminant tests (data not shown) scored +2 indicating item-scale correlations were significantly higher for the hypothesized scale than for a competing scale.

### Scale level descriptive statistics

Total subscale scores were constructed for each participant following confirmation of item scaling assumptions. Consideration was first given to the impact of select sample characteristics on subscale scores. At the second step, the properties of the subscales were examined with special attention given to the logic of mean and standard deviation scores.

#### Comparability of scale scores

It was hypothesized that subscale means should be approximately equal within the sample based on demographic and illness-related characteristics. The reader is reminded that the BK subscale is reversed scored. The *t*-test of difference and correlation tests assessed the impact of select factors on subscale scores. No significant effect was detected for carrier status, exon type, cancer presence, age or time since genetic testing (p > .05) (data not shown). However, females tended to report significantly higher levels of burden than men on the BK subscale. Women also had significantly higher mean scores than men on the FC subscale suggesting that women attach greater importance to having access to family support and resources in dealing with LS.

#### Scale properties

Subscale means, standard deviations, lowest and highest scores and score ranges were examined for both raw and transformed scores. The focus here was on the logic behind the distribution of subscale scores. For the BK subscale, a higher score is reflective of less personal and family burden associated with adjustment to hereditary cancer. Higher scores on the FC subscale are reflective of better family connectedness in dealing with the challenges posed by LS.

The pattern of mean scores and standard deviations for each subscale is summarized in Table 5. The transformed mean score (62 ± 20.9) on BK suggests that participants, on average, reported experiencing *a little to moderate* amount of burden. The transformed mean score (73 ± 19.9) on the FC subscale suggests that respondents, on average, gave high ratings to having open discussions and access to family resources/supports to handle the challenges posed by LS.

**Table 5:** **Descriptive statistics using transformed scores for Burden of Knowing (BK) and Family Connectedness (FC) scales**

Scale	Mean	SD	Range	% Missing	% At floor	% At ceiling
**BK**	62.0	20.9	0-100	9.5	0.5	0.5
**FC**	73.0	19.9	14.3-100	4.5	0.4	6.5

### Reliability and validity of PAHD

Cronbach’s alpha coefficient was used to assess internal consistency. Correlations among the subscales are useful preliminary measures of the construct validity of the entire scale. Reliability ranged from 0.83 for BK to 0.84 for FC. The reliability coefficients were above the minimum 0.70 level suggested for group level comparisons (Nunnally & Bernstein 1994). These findings suggest that the two subscales have good internal consistency.

The findings support the premise that each of the two subscales is making a distinct contribution to the overall PAHD scale. The alpha coefficients for each of the subscales are larger than the Pearson’s r values (data not shown). The subscales of the PAHD depict significant low to moderate, negative correlations with each other. That is, higher levels of family connectedness are associated with lower levels of personal and family burden in adjusting to LS.

The 243 participants provided an adequate sample for conducting factor analysis of the 17-item PAHD scale. The KMO value was 0.85 exceeding the minimally acceptable level of 0.6 (Kaiser 1970). Bartlett’s test of sphericity was also acceptable (p = 0.000), indicating the feasibility of using a factor model for the analysis. These two measures of psychometric adequacy suggested that the PAHD correlation matrix was suitable for factor analysis.

Factor analysis revealed four distinct dimensions. Based on the scree plot, it was possible to force a two-factor solution which accounted for 45.4% of the variance (Table 4). The first 10-item factor, BK, included items with loadings greater than 0.47. The scale had a reliability of 0.83. Item BK18_R appeared to be factorially complex. While its highest loading is on factor 1, it also loads on factor 2. Using ± .33 as the minimal level of practical significance for factor loadings (Ho 2006), our team could either delete the item from the analysis or rewrite it (Norman & Streiner 2008). At this stage of scale development, it was decided to retain the item for further investigation. The second 7-item factor, Family Connectedness, included items with loadings greater than 0.50. The scale had a reliability of 0.84. Overall, the factor analysis supports the qualitative and quantitative findings.

## Discussion

The PAHD scale was the outcome of a program of research that relied on survey and qualitative methods to inform the research team about psychosocial adjustment challenges in LS families. The scale was developed from content defining the struggling to adjust construct of a theoretical model generated from grounded theory. A four-member research team developed the scale by generating a large set of potential items, refining the items, and validating item content using experts and individuals from families with hereditary cancer.

By developing the PAHD from a qualitative data base, the content is steeped in the personal experiences of individuals from families with hereditary cancer. Various authors argue that instrument item-content generated from qualitative data is more likely to capture the experiences of targeted groups (Coyle & Williams 2000; McAllister 2001). It is also argued that clinical tools developed in this manner have better content and face validity and excellent psychometric properties (Gilgun 2004).

The current study provides initial evidence to support the psychometric properties of the PAHD scale. The pilot study supported the relevancy of item content and logic of the two subscale structure. Application of the MAP-R to findings from the larger sample suggests that the PAHD has acceptable internal consistency reliability, item-convergent validity and item-discriminant validity (Ware et al. 1997). Intrascale correlations compared with scale Cronbach’s alphas indicate that the two subscales (BK and FC) of the PAHD are measuring distinct but interrelated concepts.

The BK scale is intended to capture the subjective perception of individual and family burden from knowing about the presence of LS in the family. The mean BK score suggests that participants, on average, reported experiencing *a little to moderate* burden. Although no significant differences were observed for carrier and affected status or time since genetic testing, women tended to report higher levels of burden than men. Despite the limited insight from existing literature on the depth and scope of the long-term struggles of individuals living within LS families, several authors acknowledge that their complexity is shaped by the interaction of experiential cancer-based knowledge from the past and present as well as individual coping styles (Bleiker et al. 2003; McAllister 2001; d’Agincourt-Canning 2005; Kenen et al. 2003; McAllister 2002; Rolland & Williams 2005). Results from the current study support previous qualitative findings that a subgroup of individuals experience psychosocial distress in the long-term following confirmation of hereditary cancer (Watkins et al. 2011; Hamilton et al. 2009).

The second subscale, FC, is intended to capture the importance of having access to resources and family supports in sharing the burden and challenges of hereditary cancer. The mean score suggests that respondents, on average, gave high ratings to the presence of supportive family structures. Again study findings did not vary based on carrier and affected status or time since genetic testing, but women tended to value family supports more than men.

The low to moderate correlation between the two subscales support the multidimensional nature of the PAHD scale. Given that the correlations between the two scales of the PAHD are less than their reliability coefficients, there is evidence of unique reliable variance measured by each scale. A major premise of the model from which the PAHD was developed is that living in families characterized by open, supportive relationships facilitates psychosocial and emotional adjustment and decreases the burden associated with the presence of hereditary cancer. Therefore, it was expected that the subscales of the PAHD would correlate well with each other.

The findings suggest that individuals with more perceived support from family and friends tended to be less burdened from dealing with the challenges posed by hereditary cancer in the family. The value of the strength and stability of family support systems for facilitating positive coping and adjustment at the individual and family level is receiving increased attention in the research literature on genetic-based diseases (van Oostrom et al. 2003; McAllister 2001; Kenen et al. 2003; Rolland & Williams 2005).

The results of these analyses provide support for the uniqueness of the PAHD subscales and add further credence to its validity. Future studies are needed to determine the scale’s potential for monitoring the long-term psychosocial adjustment*.*

### Limitations

While the initial validation results are promising, there are a number of limitations to consider. First the study was cross-sectional and thus it is not possible to evaluate the scale’s monitoring capabilities. Second, the use of mixed methods for data collection may have influenced the findings. Further, the responders were significantly older than non-responders thus potentially limiting our knowledge of the experiences of younger individuals. Finally, it is also possible that the higher proportion of non-carriers among the non-responders may have altered the findings.

## Conclusion

The use of qualitative data to develop the PAHD has produced a scale that is steeped in the experiences of individuals and families with hereditary cancer*.* Initial testing suggests that the scale is psychometrically sound and capable of assessing psychosocial adjustment. Although study results support other findings reported in the literature, the PAHD scale is unique in that it is specific to hereditary cancer. As a clinical monitoring tool for use following genetic testing, it has the potential to identify those who are experiencing psychosocial challenges and who may require additional support for optimal adjustment.

The PAHD scale has been adapted and is being piloted in a second population with hereditary disease. A focus of this pilot is to examine the psychosocial impact of arrhythmogenic right ventricular cardiomyopathy (ARVC) on individuals and families post-genetic testing. The next stage of research for the project team will focus on implementing the PAHD scale in Community Familial Cancer Genetics Clinics throughout Newfoundland and Labrador.

## Appendix 1

Psychosocial Adjustment to Hereditary Diseases (PAHD) Scale.

We are interested in the long-term effects of a confirmed HNPCC or Lynch syndrome presence in families. Everyone goes through periods of trying to make sense of inner feelings about what the future might hold for the self and other family members. Using the scale given, you are asked to rate how well each statement reflects your situation (Table 6).

**Table 6:** **Psychosocial Adjustment to Hereditary Diseases (PAHD) Scale**

	0 Not at all	1 A little bit	2 Moderately	3 Quite a bit	4 Extremely
I think about being a carrier/non-carrier more than I should. (BK11_R)………..	**0**	**1**	**2**	**3**	**4**
I try to be positive about my future health and overall well-being. (BK12) .........	**0**	**1**	**2**	**3**	**4**
It is important for my future health not to dwell on the hereditary link to cancer in the family. (BK13) ............................................................................................	**0**	**1**	**2**	**3**	**4**
It was hard changing how often I had to screen for cancer. (BK14_R) ................	**0**	**1**	**2**	**3**	**4**
It bothers me when others do not accept my carrier/non-carrier status. (BK15_R) ..............................................................................................................	**0**	**1**	**2**	**3**	**4**
Younger people need to be encouraged to talk about all the cancer in the family. (FC16) ......................................................................................................	**0**	**1**	**2**	**3**	**4**
I find it hard dealing with younger family members who get cancer. (BK17_R)	**0**	**1**	**2**	**3**	**4**
I worry about what the future might hold for younger family members. (BK18_R) ..............................................................................................................	**0**	**1**	**2**	**3**	**4**
The stress of so much cancer in the family, more so in younger members, pulled some of us closer together but pushed others apart. (BK19_R) ................	**0**	**1**	**2**	**3**	**4**
Regular screening for cancer became a constant reminder of my cancer risk by being in this family. (BK20_R) ............................................................................	**0**	**1**	**2**	**3**	**4**
Some families handle the challenges of a strong cancer presence better than others do. We want to know how well individuals in your family support one another. Using the scale given, you are asked to rate how well each statement reflects your situation.					
Feeling supported by family and friends has helped me accept being a carrier/non-carrier. (FC21) ....................................................................................	**0**	**1**	**2**	**3**	**4**
I find it easy to seek help from family members when I need it. (FC 22) ............	**0**	**1**	**2**	**3**	**4**
It is important for everyone to talk openly about the high cancer risk in the family. (FC23) .......................................................................................................	**0**	**1**	**2**	**3**	**4**
I am concerned that the presence of hereditary cancer has hurt family relations. (BK24_R) ..............................................................................................................	**0**	**1**	**2**	**3**	**4**
I worry that all the suffering and death from cancer is placing too much burden on family members. (BK25_R) .............................................................................	**0**	**1**	**2**	**3**	**4**
Providing care to other family members with cancer has helped me become more accepting of my future. (FC26) ...................................................................	**0**	**1**	**2**	**3**	**4**
With so much cancer in the family, I worried that something would show up on my next screening test. (BK27_R) ........................................................................	**0**	**1**	**2**	**3**	**4**
When I knew there was a test to see if my family had the cancer gene, I was relieved. (FC28) ....................................................................................................	**0**	**1**	**2**	**3**	**4**
Encouragement and support from family and friends helps one accept the need for health living and cancer screening. (FC29) .....................................................	**0**	**1**	**2**	**3**	**4**

Note: R indicates items to be reverse coded. *BK* = Burden of Knowing. *FC* = Family Connectedness.
